# Variation of Sequential Ligandrol (LGD-4033) Metabolite Levels in Routine Anti-Doping Urine Samples Detected with or without Other Xenobiotics

**DOI:** 10.3390/molecules28186486

**Published:** 2023-09-07

**Authors:** Dorota Kwiatkowska, Mariola Wicka, Krzysztof Grucza, Patryk Konarski, Aleksandra Drapała, Paweł Kaliszewski

**Affiliations:** 1 Polish Anti-Doping Laboratory, Ksiecia Ziemowita 53 Bud. 4, 03-885 Warsaw, Poland; mariola.wicka@antydopinglab.pl (M.W.); krzysztof.grucza@antydopinglab.pl (K.G.); patryk.konarski@antydopinglab.pl (P.K.); aleksandra.drapala@antydopinglab.pl (A.D.); pawel.kaliszewski@antydopinglab.pl (P.K.); 2 Biological and Chemical Research Centre, University of Warsaw, Zwirki i Wigury 101, 02-093 Warsaw, Poland

**Keywords:** ligandrol, LGD-4033, VK-5211, WADA, metabolism, metabolites, steroids, SARMs, GHSs

## Abstract

Ligandrol, also known as LGD-4033, belongs to the group of selective androgen receptor modulators (SARMs). Ligandrol was first included in the WADA Prohibited List in 2018. This work presents a method that allows for the detection and identification of ligandrol and its metabolite in athletes’ urine and in dietary supplements by means of ultra performance liquid chromatography–tandem mass spectrometry (UPLC–MS/MS). Samples were prepared according to an approach involving acid hydrolysis and double liquid–liquid extraction (LLE). Furthermore, due to the lack of reference material for ligandrol metabolites, the urine collected from the control excretion study was analyzed. The presented method is appropriate to monitor ligandrol and its metabolites. The samples collected for doping control purpose contained multiple metabolites, which may potentially rule out the hypothesis of ingesting a single 1 µg or 10 µg dose only. Another aspect to take into account is that ligandrol can be applied together with SARMs, steroids, and GHSs. This will also affect the substances’ metabolism and elimination. It is also worth noting that dietary supplements may contain ligandrol as an official ingredient or as a contaminant. The described method may be usefully applied by other anti-doping or toxicological laboratories.

## 1. Introduction

It has been known for years that androgens are essential to the development and maintenance of secondary sexual characteristics in males, such as muscle and bone mass, body form, and spermatogenesis [1]. The main side effects of steroid androgen abuse are: heart attack, stroke, liver tumors, kidney failure, and psychiatric problems [2]. The discovery of selective androgen receptor modulators (SARMs; non-steroidal androgens) at the beginning of the 21st century paved the way for the possibility of their use as hormone replacement therapy alternatives to testosterone (to treat prostate, brain, and muscle conditions, as well as osteoporosis, etc.). This is due to their oral bioavailability, ease of structural modification, specificity for androgen receptors, tissue selectivity, and absence of side effects typical for anabolic steroids. However, no SARMs therapy has been approved so far. Unfortunately, SARMs can be also abused by athletes to achieve performance enhancement [3,4].

Ligandrol, also known as LGD-4033 or VK-5211, IUPAC name 4-[(2*R*)-2-[(1*R*)-2,2,2-trifluoro-1-hydroxyethyl]pyrrolidin-1-yl]-2-(trifluoromethyl)benzonitrile, belongs to the group of selective androgen receptor modulators (SARMs). Ligandrol was first included in the World Anti-Doping Agency WADA List of Prohibited Substances and Methods in 2018 under the name LGD-4033 [5]. A number of researchers have investigated the metabolism of this compound, with the aim to study its elimination and detect metabolites present in urine [6,7,8,9,10].

In 2018, Fragkaki et al. proposed ligandrol’s metabolic pathway, with chemical modifications such as epimerization, hydroxylation, oxidation, bishydroxylation, and trihydroxylation [11].

Ligandrol was originally developed as a potential treatment for preventing skeletal muscle wasting due to aging and for use in several other conditions. In sports, it can be abused both for the enhancement of muscle strength and to accelerate regeneration following physical exertion. It can be abused in combination with other substances, including testosterone, ostarine, RAD-140, GW-501516, MK677, and hGH.

The aim of this study was to develop a method capable of detecting LGD-4033 and its metabolites in athletes’ urine samples collected during doping control and dietary supplements, as well as coexisting substances listed in WADA Prohibited List [12].

## 2. Results and Discussion

**Figure 1:**
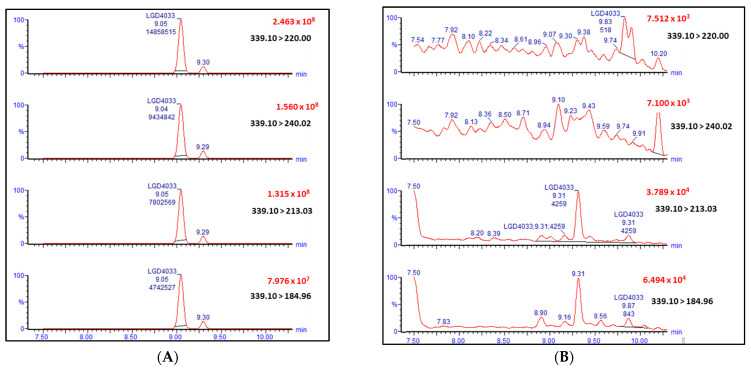
Chromatogram of the compound (LGD-4033) from the analysis of an athlete’s urine sample (**A**) and blank urine (**B**).

The LC-MS/MS method was optimized to detect LGD-4033 in the MRM reaction monitoring in positive ion mode with corresponding collision energies: *m*/*z* 220.00, *m*/*z* 240.02, *m*/*z* 213.03, *m*/*z* 184.96 and 30 eV, 25 eV, 40 eV, and 40 eV, respectively. Figure 1 shows a typical chromatogram for LGD-4033 in a real sample.

Relative extraction efficiency for LGD-4033 at a final concentration of 2 ng/mL using six different urine samples ranged from 79.7% to 99.15%; (Table 1).

Specificity was determined based on results for 16 blank urine samples collected from different individuals. All selected ion transitions for the target compound proved to be specific, as no interfering matrix-based signals were observed for each MRM in the analysis of chromatograms of blank urine samples.

Analyte carry-over was assessed based on the injection of a urine sample spiked with the standard of the analyzed substance with the highest final concentration (10 ng/mL) for six different blank samples. The results were evaluated by analyzing chromatograms obtained in the process. Signal-to-noise (S/N) Ratio: magnitude of the instrument response to the analyte (signal) relative to the magnitude of the background (noise) was defined as acceptable when its value was above 3 [13]. The sample’s carry-over was not observed.

Linearity was determined at nine concentration levels within the 0.05 ng/mL to 10 ng/mL range, using six urine samples at differing pH levels, specific gravity, and sex of donor. pH values of urines used ranged from acidic through neutral to basic, at 5.61 to 7.04, while specific gravity varied between 1.005 g/mL and 1.020 g/mL.

Calibration curves were linear for the range from 0.050 ng/mL to 10 ng/mL for the target compound in six different matrices, as shown in Table 2. The acceptance criterion adopted was the coefficient of determination of at least r^2^ ≥ 0.950.

LOD and LOQ values obtained are presented in Table 3. LOD was found by analyzing each substance in the matrix with decreasing concentrations over two repetitions in six measurement series. LOQ was determined based on the slope of the calibration curve and was established at 3.3 × SD/a.

Intra-study accuracy and short-term precision of the method (measurements performed over a single day) were determined for six concentration levels, 0.50 ng/mL, 0.75 ng/mL, 1.0 ng/mL, 2.0 ng/mL, 5.0 ng/mL, and 10 ng/mL, for the compound of interest over six repetitions. Accuracy was calculated as the percentage difference between the measured concentration and the calculated con-centration of the standard added divided by the calculated concentration of the standard added. Precision was expressed as %RSD (coefficient of variation), calculated as percentage standard deviation divided by the mean of measured concentration values. Summary statistics of intra-day accuracy and precision are provided in Table 4.

### 2.1. LGD-4033 Metabolites

**Figure 2:**
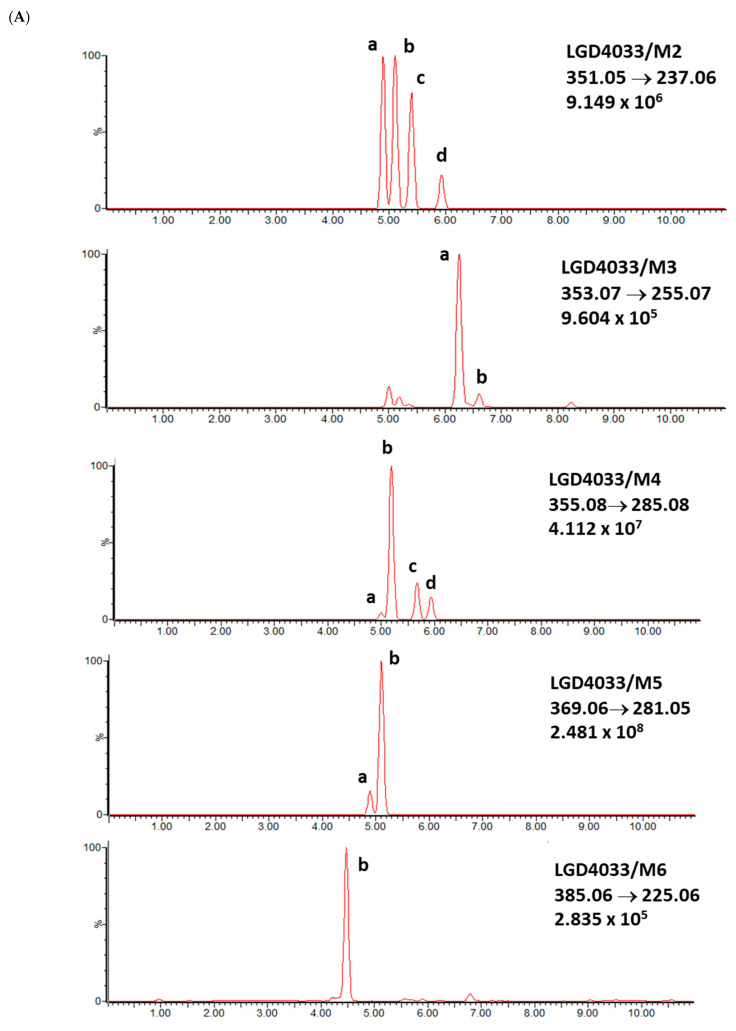

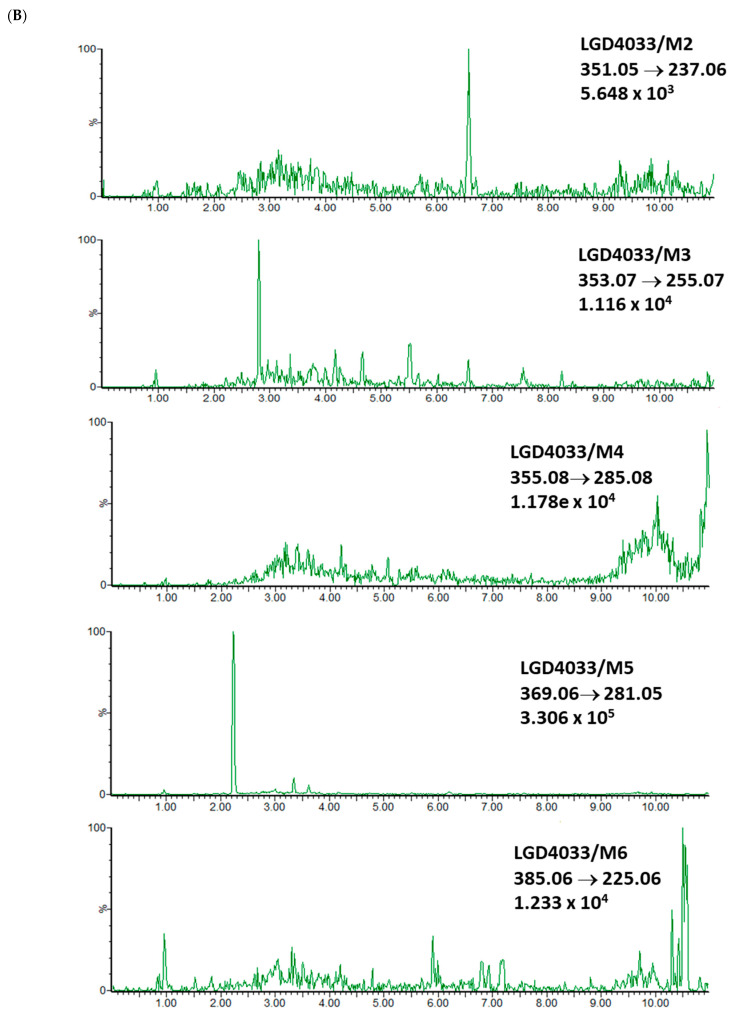
Exemplary chromatograms of the analyzed metabolites of ligandrol (LGD-4033) in a real urine sample (**A**) and in blank urine (containing no target substance) (**B**). The symbols ‘a, b, c, d’ denote different spatial orientations of substituents, as shown in Table 5.

Identification of the ligandrol metabolites was conducted using urine reference material [14] and the results presented in Figure 2.

### 2.2. Real Samples and Other Prohibited Substances in Sport

Five routine anti-doping samples were confirmed to contain LGD-4033 and its metabolites. They were collected in-competition from male athletes competing in strength sports such as: weight lifting, bouldering, canoeing, and powerlifting.

To identify ligandrol and its metabolites in the athletes’ urine samples, the CRM of ligandrol and reference urine sample were used, respectively. The estimated concentrations of ligandrol ranged from 1.4 ng/mL to 545 ng/mL. In turn, the number of identified metabolites of ligandrol ranged from 8 to 13 (Table 6 and Table 7).

**Figure 3:**
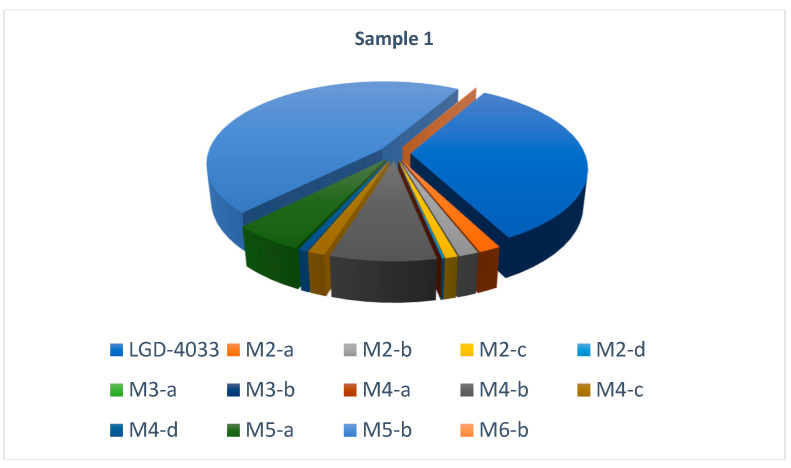

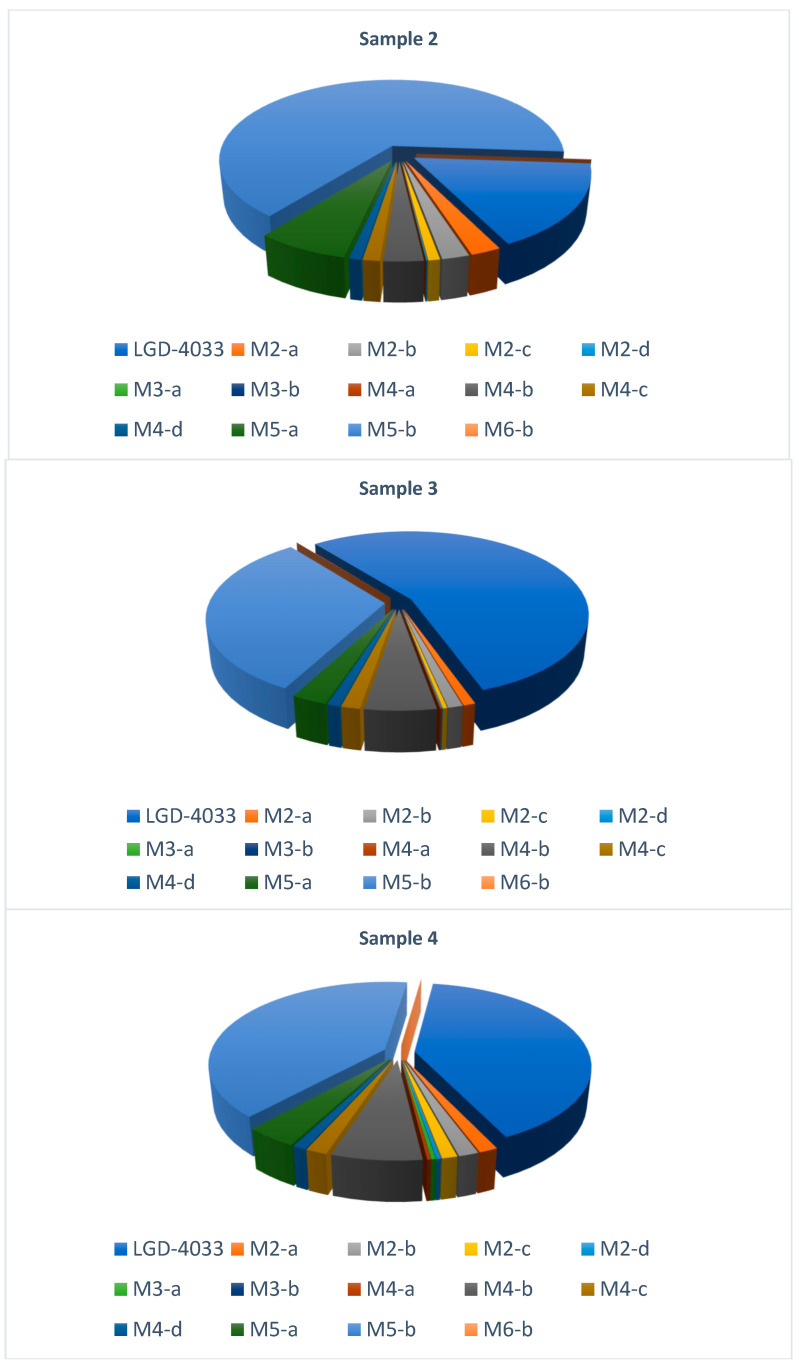

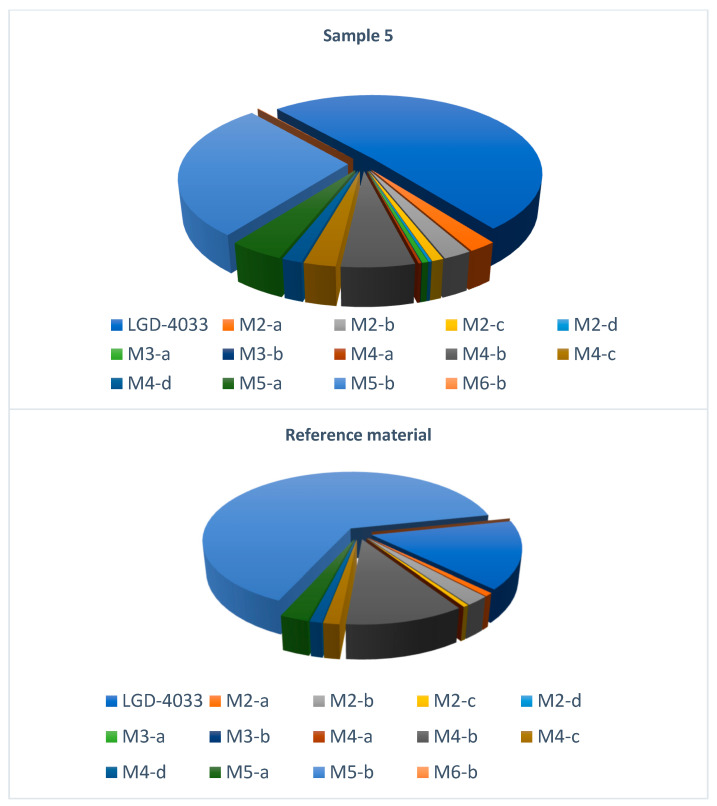
Distribution of detected metabolites and parent compound vs. the internal standard.

The distribution of observed metabolites and parent compound vs. the internal standard is shown in Figure 3.

According to the findings, the M5-b metabolite was present at the highest relative abundance in all samples with LGD-4033 detected. The next two in terms of relative abundance were M4-b and M5-a. As such, M4-b, M5-a, and M5-b appear to be the best candidates for monitoring.

Additional substances were detected in samples 1, 3, and 5. The details are shown in Table 8.

Arimistane is an aromatase inhibitor belonging to the group of hormones and metabolic modulators. The purpose of combining it with LGD-4033 may be to restore hormonal balance in individuals with excessive levels of estrogens in the body.

Ibutamoren belongs to peptide hormones and their releasing factors as a growth hormone secretagogue (GHS). The combination of ibutamoren and LGD-4033 increases muscle mass.

Ostarine, like LGD-4033, is classified among other anabolic agents. Combining these two substances increases fat-free body mass and muscle mass.

Dehydrochlormethyltestosterone and nandrolon belong to anabolic androgenic steroids (AAS). The combination of LGD-4033 and AAS increases muscle mass.

RAD140 is also a substance belonging to the same group as LGD-4033, i.e., anabolic agents, but to the so-called other anabolic agents. It helps to increase muscle mass. The combination of these two substances potentiates their effects.

As the above list demonstrates, it was possible to combine compounds from different classes having divergent effects.

### 2.3. Analysis of Dietary Supplements for the Purposes of Disciplinary Proceedings

One originally packed and one opened dietary supplement with different LOT numbers were delivered by the POLADA to the Warsaw anti-doping laboratory after reporting an AAF result for ligandrol. On one of them, the ligandrol was mentioned on the label as an official ingredient.

**Figure 4:**
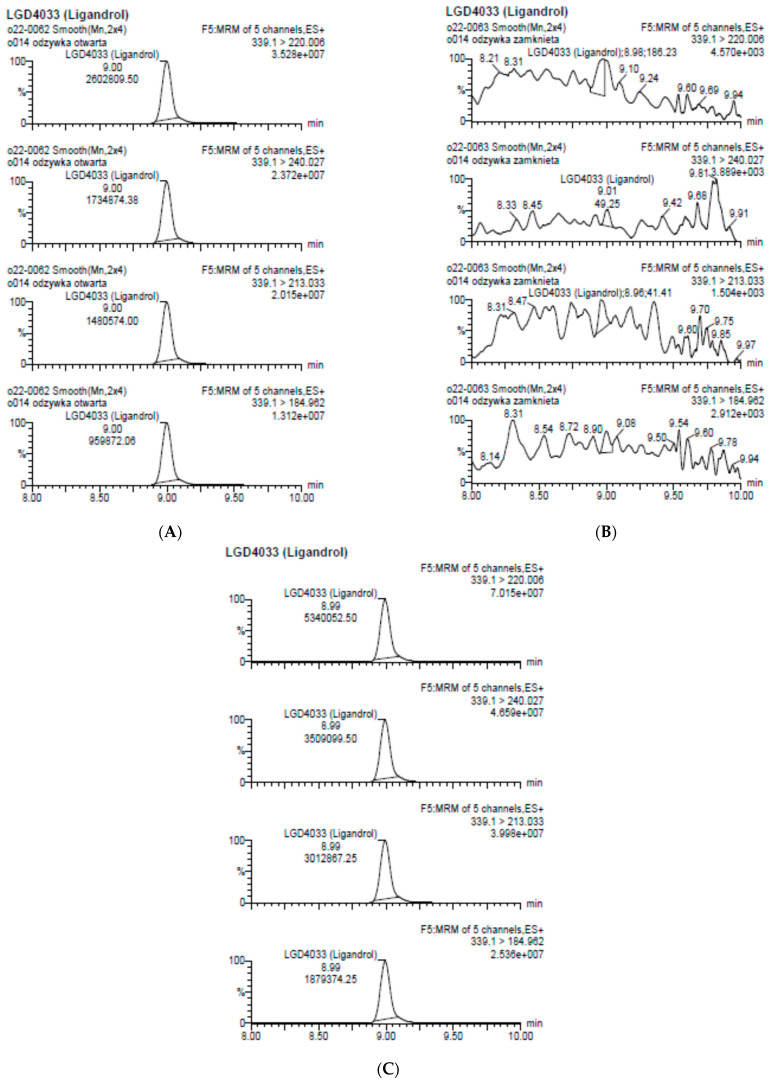
Chromatograms for dietary supplement (**A**) (containing ligandrol), (**B**) (without ligandrol), and (**C**) (spiked control sample with ligandrol).

The performed analysis showed the presence of ligandrol in one of them (Figure 4).

## 3. Materials and Methods

### 3.1. Chemical and Materials

The standard of ligandrol (LGD-4033) was purchased from Toronto Research Chemicals; TRC (Toronto, ON, Canada). Stock solution of standard substance was prepared at the concentration of 1 mg/mL and its working solutions (100 µg/mL, 10 µg/mL, and 1 µg/mL) in methanol and stored at −20 °C.

Due to unavailability of certified reference materials of LGD-4033 metabolites, the reference urine collected from volunteers who ingested 50 µg of LGD-4033 dissolved in 120 mL of drinkable yogurt was utilized in the experiment [14].

LC/MS-grade of methanol and acetonitrile were purchased from MerckMillipore (Darmstadt, Germany) and Fisher Chemical (Hampton, NH, USA), respectively. Disodium phosphate and monosodium phosphate ≥ 99% purity were supplied by Honeywell (Charlotte, NC, USA), while potassium carbonate and potassium bicarbonate, both HPLC grade, were purchased at POCH S.A. (Gliwice, Poland). In turn, β-glucuronidase (*Escherichia coli*) and HPLC-grade methyl tert-butyl ether were supplied by Roche (Basel, Switzerland) and J.T.Baker (Philipsburg, NJ, USA), respectively. The Millipore DirectQ UV3 system (Darmstadt, Germany) was used as the source of water (R > 18 MΩ/cm). Sample preparation of dietary supplement samples followed the protocol described in [19,20].

### 3.2. Sample Preparation

#### 3.2.1. Urine Samples

Samples for analysis were stored frozen. Before analyzing, samples were allowed to reach room temperature.

Urine samples were prepared according to the protocol described previously [21], i.e., 3 mL of urine was spiked with internal standards. After addition of 1.1 mL of 1 M of 6.5 pH phosphate buffer and 50 µL of β glucuronidase (*E. coli*), the samples were incubated at 50 °C for 65 min. Afterward, the samples were cooled down to room temperature. Next, after an addition of 1 mL of 20% K_2_CO_3_/KHCO_3_ (1:1, *v*/*v*), the extraction with 6 mL of methyltert-butyl ether was performed (20 min). Samples were then centrifuged (5 min/16,495 RCF), and the ether phase was recovered and evaporated under a nitrogen flow at 55 °C. The dry residue was reconstituted in 100 μL of acetonitrile/H_2_O mixture (1:1, *v*/*v*).

#### 3.2.2. Dietary Supplements

Dietary supplement samples were prepared according to the protocol described previously [19,20].

### 3.3. Instrumental Analysis

#### 3.3.1. Liquid Chromatography

Analysis was performed on a UPLC Acquity chromatograph (Waters, Milford, MA, USA) equipped with BEH C18 column (1.7 μm, 2.1 × 100 mm). The mobile phase consisted of 0.1% formic acid in acetonitrile (A) and 0.1% formic acid in water (B) and the LC gradient was employed at the constant flow rate of 300 μL/min at 45 °C. The following gradient flow rates were used: 0–2 min, 5% (A) + 95% (B); 2–8 min, 35% (A) + 65% (B); 8–9 min, 50% (A) + 50% (B); 9–10 min, 100% (A); 10–11 min, 100% (A); 11 min.

#### 3.3.2. Mass Spectrometry

Multiple reaction monitoring (MRM) of the studied substances was conducted using an XevoTQ-XS (Waters, Milford, MA, USA) mass spectrometer equipped with an electrospray ionization (ESI) source, which operated in the positive ion mode (ESI (+)) and negative ion mode (ESI (−)). The desolvation gas flow was set at 800 L/h at 600 °C and the source temperature was 150 °C. The applied capillary voltage was 3.0 kV. In turn, the collision gas flow was set at 0.20 mL/min.

Traced MRMs and their corresponding MS settings are listed in Table 5.

#### 3.3.3. Assay Characterization

The following analytical parameters were evaluated for ligandrol: linearity, limit of detection (LOD), and limit of quantitation (LOQ). In addition, relative extraction efficiency was determined. Selectivity, analyte carry-over, robustness control, and intra-test precision, and consistency were also determined. Due to the lack of certified reference material for ligandrol metabolites, their identification was conducted using reference urine.

#### 3.3.4. Linearity, LOD, LOQ

The linearity of the method was determined by analyzing two independent measurements for ligandrol, which was fortified to the six blank urine samples at the concentrations of 0.05 ng/mL, 0.1 ng/mL, 0.25 ng/mL, 0.5 ng/mL, 0.75 ng/mL, 1 ng/mL, 2 ng/mL, 5 ng/mL, and 10 ng/mL.

The blank urine samples used differed in pH values and specific gravity, and were collected from three males and three females. pH values ranged from 5.61 to 7.04, while specific gravity was between 1.005 g/mL and 1.020 g/mL. The acceptance criteria adopted was the curve coefficient of determination (r^2^) of at least ≥0.95. Measurements were performed on different days. The method’s limit of detection (LOD) for the compound analyzed was determined with the use of blank urine and urines spiked with LGD-4033 at 0.05 ng/mL, 0.10 ng/mL, 0.25 ng/mL, 0.50 ng/mL, 0.75 ng/mL, 1.0 ng/mL, 2.0 ng/mL, 5.0 ng/mL, and 10 ng/mL. LOD was determined based on the slope of the calibration curve and found to be 3.3 × SD/a, where ‘a’ is the slope of the curve. The limit of quantification (LOQ) was estimated at 10 × SD/a, where ‘a’ is the slope of the curve.

#### 3.3.5. Relative Extraction Efficiency

Extraction efficiency was determined by analyzing two sets of samples spiked with ligandrol at 2 ng/mL using six different blank urine samples. Type C samples (*n* = 6) were obtained by spiking with ligandrol and ISTD prior to solid-phase extraction. Type B samples (*n* = 6) were prepared by spiking with analyte and ISTD at concentrations equal to type C samples following extraction. Relative extraction efficiency (RE) was calculated as: RE (%) = (C/B) × 100, where ‘B’ and ‘C’ are ratios of ligandrol peak area to the internal standard peak area.

#### 3.3.6. Selectivity, Sample Carry-Over, and Method Robustness Control

Selectivity of the method was assessed via the analysis of 16 urine samples collected from volunteers. For each, a blank urine sample and one spiked with ligandrol at the concentration of 10 ng/mL were compared. Evaluation of the chromatograms at the retention time of ligandrol showed the absence of any interfering components. Moreover, the ratio of signal to noise (S/N) above 3:1 was observed, which meets WADA criteria [13]. Carry-over was evaluated through the injection of one blank urine sample directly after the sample spiked with ligandrol at 20 ng/mL. The presence of carry-over was evaluated via the visual inspection of the chromatogram obtained for the blank sample and which revealed no noticeable carry-over. Method robustness was verified by analyzing control samples prepared on at least the same analysis day from at least six different urine samples. Observed variable parameters included, among others, the number of technicians preparing the samples, specific reagent batches, and degree of device contamination.

#### 3.3.7. Intra-Laboratory Repeatability and Reproducibility of the Method

Precision was evaluated on the basis of standard deviation calculated for a series of measurements, while accuracy was assessed based on the magnitude of standard error determined on the results obtained for 6 different samples in a measurement series at six distinct concentration levels: 0.50 ng/mL, 0.75 ng/mL, 1.0 ng/mL, 2.0 ng/mL, 5.0 ng/mL, and 10 ng/mL.

## 4. Conclusions

The method presented in this paper allows for separating and monitoring ligandrol and its metabolites in urine and dietary supplement samples. Elimination involves various metabolic transformations; however, based on the reference material, i.e., urine process collected from control exertion study and athletes’ urine samples provided to the laboratory, it seems appropriate to monitor ligandrol as parent compound and metabolites following hydroxylation and ring cleavage for the M4-b metabolite and bis-hydroxylation for the M5-a and M5-b metabolites. Further studies are required, comprising syntheses of those three metabolites, in order to confirm their proposed structure. Moreover, it would allow for their unequivocal identification and estimation of their concentrations.

Wagener et al. [14] showed LGD-4033 metabolite excretion timelines depending on the dose and frequency of application. They tried to establish the time of administration, dose, and the source of ingestion (such as a dietary supplement contaminated with ligandrol) by analyzing the presence of different metabolites. They assumed that if the ratio M1/LGD-4033 was 0.12 (the M1 metabolite, the epimer of LGD-4033 was not observed in the present study) and LGD-4033 concentration was 0.2 ng/mL, and if the athlete had used the supplement resulting in a daily intake dose of 10 ug, then the ingestion may have occurred unintentionally between 4 and 30.5 h earlier.

However, in the case of routine samples of which analytical findings were presented in the study, this assessment may be incomplete due to the fact we did not know the dose, how often the substance was used, and how other xenobiotics affect LGD-4033 elimination and metabolism. The concentration of ligandrol in the samples tested ranged from 1.4 ng/mL to 545 ng/mL.

It seems worthwhile to consider time of ingestion or dose. According to Wagener et al. [14], if the ingested dose is 1 µg, the M2-d, M3-b, and M4-a metabolites are not found, while M1 and M2-c may be present. The M2-d, M3-b, and M4-a metabolites are seen with an ingestion of a single 10 µg dose. A single 50 µg dose results in the presence of all metabolites.

The picture is different with multi-doses. Here, according to the authors, 5 days of dosing results in accumulation, but still, there are no M3-b or M4-a metabolites seen with the lowest dose. For M1, M2-c, M2-d, and M6-a, and with a dose of 10 µg or 50 µg, all metabolites are detected, with M5-b lasting longest (still present at 1000 h).

As for the samples collected for doping control, all of them contained the M2-d metabolite, which may potentially rule out taking a single 1 µg or 10 µg dose, since all or almost all of the metabolites used in the method were detected in the samples. Another piece of evidence suggesting that we are dealing with multiple doses in high concentrations is the concentration of LGD-4033. According to the literature, following an intake of 50 µg dose for 5 days, the values were not higher than 6.4 ng/mL. Out of the real samples, only one had a lower concentration of 1.4 ng/mL but with the presence of nine metabolites, suggesting that LGD-4033 intake may have occurred some time before.

Another aspect to take into account is that, just as anabolic steroids may be combined with, for example, other anabolic steroids, diuretics, and SARMs, so can ligandrol be taken together with other steroids, SARMs, and GHSs. This will also affect the substances’ metabolism and elimination.

It is also worth noting that dietary supplements may contain ligandrol as an official ingredient. The one tested here listed ligandrol on the label. It should be noted that ligandrol, a prohibited substance in sports, may be present in dietary supplements as a contaminant. This phenomenon is very well known in the anti-doping community and is often presented in papers [19,20].

The presented data fully support the usefulness of the developed method for anti-doping or toxicological investigations. The detection of multiple metabolites evidence substantially that the LGD4033 has been applied and can serve as more reliable proof for proceedings.

## Figures and Tables

**Table 1:** Extraction efficiency for LGD-4033 in urine ^1^.

Compound	RE (%) (Mean)	Precision (%RSD)
LGD-4033	89.91	7.40

^1^ Data in the table are the mean value from *n* = 4 measurements at 15 ng/mL.

**Table 2:** Calibration data for LGD-4033.

Compound	Urine						
	1C	2C	3C	4C	5C	6C	%RSD
	R^2^						
LD-4033	0.9993	0.9978	0.9996	0.9943	0.9982	0.9969	0.19

*n* = 2—each value is the mean of two measurements. C stands for individual curve obtained.

**Table 3:** LOD and LOQ values obtained.

Substance	LOD (ng/mL)	LOQ (ng/mL)
LGD-4033	0.5	0.80

**Table 4:** Short-term accuracy and precision of LGD-4033 measurements.

	Measurements over One Day		
Compound	Concentration (ng/mL)	Precision (%RSD)	Accuracy (%)
LGD-4033	0.500	10.73	8.33
	0.750	5.96	0.00
	1	4.29	2.50
	2	2.12	1.25
	5	5.72	2.50
	10	1.21	0.33

**Table 5:** Analyzed MRMs for the target compound and its metabolites [14].

Analyte	Proposed Chemical Structure	Metabolic Transformation	Precursor Ion (*m*/*z*) [M]^+^ Parent Compound and [M]^−^ Metabolites	Product Ion (*m*/*z*)	Retention Times (min) Determined Experimentally
LGD-4033	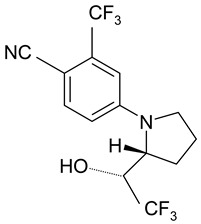		339.10	220.00; 240.02; 213.03; 184.96	9.05
M2-a	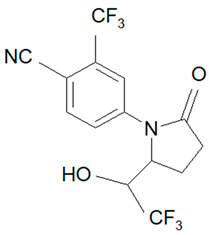	Hydroxylation, Dehydrogenation	351.05	237.06; 253.02; 281.05	4.90
M2-b				237.06; 253.02; 281.05	5.11
M2-c				237.06; 253.02; 281.05	5.40
M2-d				237.06; 253.02; 281.05	5.93
M3-a	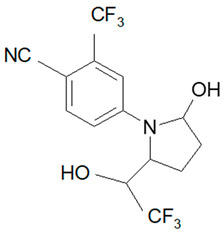	Hydroxylation	353.07	255.07; 199.04; 185.03	6.25
M3-b				255.07; 185.03	6.40
M4-a	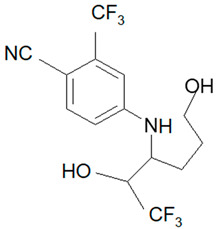	Hydroxylation, Ring cleavage	355.08	285.08; 257.09; 185.03	5.00
M4-b M4-c				285.08; 257.09; 185.03	5.19
				285.08; 185.03	5.67
M4-d				285.08; 185.03	5.93
M5-a	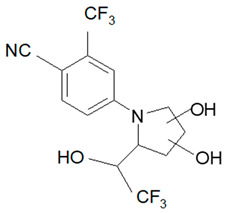	bis-Hydroxylation	369.06	281.05; 237.06; 253.05	4.89
M5-b				281.05; 237.06; 255.07	5.10
M6-b	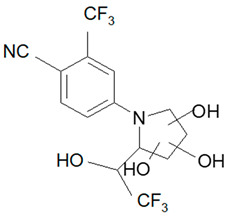	tri-Hydroxylation	385.06	225.06; 227.04	4.48

**Table 6:** The presence of LGD-4033 and its metabolites (LGD-4033 concentration estimated in ng/mL; the presence of metabolites based on reference urine).

Analyte	Sample 1	Sample 2	Sample 3	Sample 4	Sample 5
**LGD-4033**	45.5	1.45	324	125	545
**M2-a**	+	+	+	+	+
**M2-b**	+	+	+	+	+
**M2-c**	+	+	+	+	+
**M2-d**	+	+	+	+	+
**M3-a**	-	-	-	+	+
**M3-b**	-	-	-	-	+
**M4-a**	-	-	+	+	+
**M4-b**	+	+	+	+	+
**M4-c**	+	+	+	+	+
**M4-d**	+	+	+	+	+
**M5-a**	+	+	+	+	+
**M5-b**	+	+	+	+	+
**M6-b**	+	-	+	+	+

+ detected; - not detected.

**Table 7:** Ratios of target compounds (TCs) vs. parent compound and Epitestosterone-d3 internal standard (ratios of areas under the peaks).

Analyte	Sample 1		Sample 2		Sample 3		Sample 4		Sample 5		Reference Material	
	TC/PC	TC/ISTD	TC/PC	TC/ISTD	TC/PC	TC/ISTD	TC/PC	TC/ISTD	TC/PC	TC/ISTD	TC/PC	TC/ISTD
**LGD-4033**	1	1.7625	1	0.1498	1	28.0490	1	6.8591	1	101.9872	1	0.0831
**M2-a**	0.0594	0.0886	0.1624	0.0213	0.0199	0.4629	0.0434	0.2437	0.0756	4.5054	0.0514	0.0042
**M2-b**	0.0534	0.0796	0.1464	0.0192	0.0246	0.5727	0.0462	0.2594	0.0764	4.5539	0.1406	0.0116
**M2-c**	0.0338	0.0504	0.0628	0.0082	0.0075	0.1734	0.0358	0.2012	0.0313	1.8633	0.0330	0.0027
**M2-d**	0.0069	0.0103	0.0073	0.0010	0.0023	0.0539	0.0106	0.0595	0.0086	0.5103	-	-
**M3-a**	-	-	-	-	-	-	0.0109	0.0610	0.0168	1.0008	-	-
**M3-b**	-	-	-	-	-	-	-	-	0.0010	0.0621	-	-
**M4-a**	-	-	-	-	0.0020	0.0458	0.0082	0.0462	0.0079	0.4706	-	-
**M4-b**	**0.2818**	**0.4205**	0.2131	0.0279	**0.1189**	**2.7653**	**0.2082**	**1.1704**	**0.1968**	**11.7233**	**0.6973**	**0.0574**
**M4-c**	0.0467	0.0697	0.0918	0.0120	0.0317	0.7381	0.0492	0.2766	0.0865	5.1555	0.0943	0.0078
**M4-d**	0.0245	0.0365	0.0643	0.0084	0.0216	0.5023	0.0303	0.1704	0.0536	3.1921	0.0718	0.0059
**M5-a**	0.1902	0.2838	**0.4915**	**0.0644**	0.0588	1.3668	0.1207	0.6784	0.1479	8.8091	0.1684	0.0139
**M5-b**	**1.5928**	**2.3765**	**4.5244**	**0.5928**	**0.6918**	**16.0886**	**1.2134**	**6.8203**	**0.9451**	**56.3030**	**4.2050**	**0.3463**
**M6-b**	0.0031	0.0047	-	-	0.0011	0.0256	0.0014	0.0081	0.0035	0.2115	-	-

**Table 8:** Other substances detected in samples with LGD-4033.

Compounds Detected	Estimated Concentration (ng/mL)
Sample 1	
Arimistane (Androst-3,5-diene-7,17-dione) [15]	110.65
Arimistane M (Androst-3,5-diene-7β-ol-17-one) [16]	454.38
Ibutamoren (MK-677)	44.46
Hydroxyibutamoren M1 [17]	not determined
Hydroxyibutamoren M2 [17]	not determined
Dihydroxyibutamoren M3 [17]	not determined
Desbenzylibutamoren M4 [17]	not determined
Ostarine	924.42
o-Dephenylostarine	11.14
Hydroxyostarine M1a [18]	not determined
Hydroxyostarine M1b [18]	not determined
Sample 3	
Ibutamoren (MK-677)	0.13
Sample 5	
Ibutamoren (MK-677)	203.25
Dehydrochlormethyltestosterone M3 (4-chloro-18-nor-17β-hydroxymethyl,17α-methyl-5α-androst-13-en-3α-ol)	1.1
LGD-4033	544.86
LGD-4033 M5a (bis-hydroxylation)	not determined
Ostarine	1.03
RAD140	844.97
Nandrolone M (19-norandrosterone)	3.71

## Data Availability

Not applicable.
